# Expression, Localization and Prognosis Association of MEP50 in Breast Cancer

**DOI:** 10.3390/cancers14194766

**Published:** 2022-09-29

**Authors:** Samyuktha Suresh, Mathilde Vinet, Rayan Dakroub, Laetitia Lesage, Mengliang Ye, Hussein Fayyad-Kazan, André Nicolas, Didier Meseure, Thierry Dubois

**Affiliations:** 1 Breast Cancer Biology Group, Translational Research Department, Institut Curie-PSL Research University, 75005 Paris, France; 2 Laboratory of Cancer Biology and Molecular Immunology, Faculty of Sciences-I, Lebanese University, Hadath 1003, Lebanon; 3 Platform of Experimental Pathology, Department of Diagnostic and Theranostic Medicine, Institut Curie-Hospital, 75005 Paris, France

**Keywords:** triple-negative breast cancer, TNBC, breast cancer, PRMT5, MEP50, H4R3me2s, epigenetics, prognosis, recurrence-free survival

## Abstract

**Simple Summary:**

The arginine methyltransferase PRMT5 is an emerging therapeutic target for various cancers including breast cancer. In this study, we examine the expression and subcellular localization of its main cofactor, MEP50, in the different breast cancer subgroups. High levels of MEP50 are found in TNBC and associated with better recurrence-free survival.

**Abstract:**

Breast cancer is composed of distinct subgroups, triple-negative breast cancer (TNBC), human epidermal growth factor receptor-2 (HER2), luminal A, and luminal B, which are associated with different prognosis. MEP50 is the main partner of the arginine methyltransferase PRMT5 required for its enzymatic activity. Here, we examined *MEP50* expression in the different breast cancer subgroups from the transcriptomic data obtained on human breast cancer samples and on normal breast tissues in two cohorts (Curie, *n* = 141; The Cancer Genome Atlas—TCGA, *n* = 788). We observed higher levels of *MEP50* mRNA in TNBC (Curie, *n* = 41; TCGA, *n* = 106) compared to the other breast cancer subgroups and normal breast tissues. Using an online KM-plotter database, which allows survival analyses in a larger number of breast cancer patients, we found that high *MEP50* mRNA levels were associated with a more favorable recurrence-free survival (RFS) in TNBC (*n* = 953, *p* = 1.2 × 10^−4^) and luminal B (*n* = 1353, *p* = 0.013) tumors, whereas high *PRMT5* mRNA levels were associated with worse RFS in these two subgroups (TNBC: *n* = 442, *p* = 1.0 × 10^−^^4^; luminal B: *n* = 566, *p* = 6.8 × 10^−3^). We next determined the expression and the subcellular localization of MEP50 protein by immunohistochemistry (IHC) in our Curie cohort of breast cancer (*n* = 94) and normal tissues (*n* = 7) using a validated MEP50 antibody. MEP50 was more expressed in breast tumors compared to normal breast tissues (*p* = 0.02). MEP50 was more localized to the cytosol in breast cancer cells compared to normal breast tissue (*p* = 4 × 10^−4^), and was more found at the plasma membrane in normal tissues compared to breast tumors (*p* = 0.01). We also evaluated PRMT5 activity by IHC in our Curie cohort using a validated antibody (H4R3me2s) detecting histone H4 symmetrically dimethylated on Arg3. High levels of H4R3me2s were found in normal breast tissues, whereas the lowest levels of H4R3me2s were observed in TNBC and HER2 breast cancer subgroups. Altogether, our study reports the expression of the PRMT5 cofactor (MEP50) and substrate (H4R3me2s) in breast cancer and highlights the association of *PRMT5* and *MEP50* mRNA with prognosis in luminal B and TNBC breast cancer subgroups and certain TNBC subtypes.

## 1. Introduction

Breast cancer is a heterogenous disease comprising several subgroups associated with different prognosis [1]. Breast tumors are mainly classified depending on the expression of hormone receptors (estrogen and progesterone receptors, ER and PR) and the overexpression of epidermal growth factor receptor 2 (HER2) [1]. Luminal breast cancers express ER and/or PR, and are subclassified into luminal A or luminal B subgroups, the former being less proliferative and associated with a better prognosis. Hormone-negative breast cancers, which are associated with the poorest prognosis, are subdivided into two groups depending on HER2 overexpression: HER2-positive (HER2), characterized by *HER2* amplification, and basal-like (or triple-negative breast cancer, TNBC) [1]. TNBC itself is highly heterogeneous comprising molecularly distinct subtypes: basal-like 1 (BL1), basal-like 2 (BL2), mesenchymal (M), mesenchymal stem-like (MSL), immunomodulatory (IM), and luminal androgen receptor (LAR) [2,3,4]. This inter-tumor heterogeneity along with a well-established intra-tumor heterogeneity arising from drug-resistant cells pose a major challenge in treating TNBC patients [2,3,4,5,6]. Identifying new therapeutic targets to overcome chemo-resistance and recurrence is a high clinical priority for TNBC patients.

Protein arginine methyltransferases (PRMT1-9) are post-translational modifying enzymes which transfer one or two methyl group(s) to a wide range of cytosolic and nuclear substrates [7,8,9,10,11,12]. Some PRMTs are emerging as attractive therapeutic targets as they have been shown to be overexpressed in various cancers. We have recently reported that PRMT1 [13] and PRMT5 [14] are promising targets for TNBC. Specific PRMT5 inhibitors are currently under evaluation in clinical trials [8,9,15,16]. 

PRMT5 regulates gene expression through transcriptional activation and repression, pre-mRNA splicing, translation, growth factor signaling, and DNA damage response, to name a few [16,17,18]. PRMT5 is the principal enzyme catalyzing symmetric dimethylation of arginine on a myriad of substrates including histones (H2A, H3 and H4), and non-histone proteins. Symmetric dimethylation of arginine 3 on histone H2A (H2AR3me2s) [19] and H4 (H4R3me2s) [20,21] and arginine 8 on histone H3 (H3R8me2s) [21,22] are associated with gene regulation. 

The main protein partner of PRMT5 is the methylosome protein 50 (MEP50) [23]. It is also known as WDR77 (WD repeat-containing protein) or as a coactivator of the androgen receptor (p44) [24]. MEP50 forms a hetero-octameric complex with PRMT5 and activates its enzyme activity [25,26,27]. MEP50 is overexpressed in lung, squamous cell carcinoma, and breast cancer at the RNA level [28,29,30] and in ovarian [31], lung [32], and squamous cell [33] carcinomas at the protein level. Mutations in the *MEP50* gene that impairs its binding to PRMT5 have been discovered in familial non medullary thyroid cancer [34]. High *MEP50* mRNA levels are associated with poor prognosis in lung [17] and breast [35] cancers. MEP50 localizes both in the cytosol and nucleus of various cancer cells: breast [29,36], ovarian [31], squamous [33], and prostate [37,38,39,40,41] cancers. Cytosolic localization of MEP50 is associated with proliferation while nuclear MEP50 is linked with differentiation in prostate cancer cells [37,38,39,40,41,42]. Knocking down MEP50 decreases cell proliferation of different cell lines including ovarian cancer [31], squamous cell carcinoma [33], keratinocyte [43], and lung cancer [44] cells. In contrast, its knockdown increases thyroid cancer cell growth [34]. A recent study showed that MEP50 depletion sensitizes prostate cancer cells to radiation [45].

In this study, we examined the expression level of *MEP50* and *PRMT5* mRNA and their association with recurrence-free survival (RFS) in the different breast cancer subgroups and various TNBC subtypes. We determined the expression and the subcellular localization of MEP50 protein by immunohistochemistry (IHC) in our cohort of breast cancer tissues. Lastly, we assessed nuclear PRMT5 activity by analyzing the H4R3me2s methylation mark by IHC in breast tumors and normal tissues. 

## 2. Materials and Methods

### 2.1. Human Breast Cancer Cohorts and Transcriptomic Data

Curie cohort: Our cohort has been previously described [14,46,47,48,49] and is composed of TNBC (ER-, PR-, HER2-), HER2 (ER-, PR-, HER2+), luminal A (ER+ and/or PR+, HER2-), luminal B (ER+ and/or PR+, HER2+), and normal breast tissues from plastic surgery. Experiments were conducted in accordance with Bioethics Law No. 2004–800 and the Ethics Charter from the French National Institute of Cancer (INCa), and after approval from the ethics committee of our Institution. Transcriptome microarray (U133 Plus 2.0 Affymetrix chips, Thermo Fisher Scientific, Waltham, MA, USA) was performed on TNBC (*n* = 41), HER2 (*n* = 30), luminal A (*n* = 29), luminal B (*n* = 30), and normal human samples (*n* = 11), as previously described [14,46,47,48,49] (Table 1).

TCGA cohort: the publicly available RNA-SeqV2 Level 3 dataset (January 2015) were downloaded from The Cancer Genome Atlas (TCGA) breast invasive carcinoma cohort (http://cancergenome.nih.gov/) [50] and integrated into a platform in knowledge data integration (KDI) at Institut Curie (https://bioinfo-portal.curie.fr). We classified the breast cancer subgroups, as we did for the Curie cohort, based on the immunohistochemical status for ER, PR and HER2 which were provided in the dataset. TNBC (ER-, PR-, HER2-; *n* = 106), HER2 (ER-, PR-, HER2+; *n* = 36), luminal A (ER+ and/or PR+, HER2-; *n* = 415), and luminal B (ER+ and/or PR+, HER2+; *n* = 118). The TCGA database includes 113 referenced normal breast tissue samples (Table 2).

### 2.2. Survival Analysis

Kaplan–Meier curves for target genes were generated with the online tool Kaplan–Meier Plotter (KM) plotter (https://www.kmplot.com, accessed on August 2022) [51]. The best probe sets for MEP50 (201421_s_at) and PRMT5 (1564520_s_at) retrieved the number of patients for survival analyses (Table 3 and Table 4 indicate the number of patients analyzed in each breast cancer subgroup and TNBC subtype, respectively). Recurrence-free survival (RFS) of breast cancer patients stratified by high and low expression of *MEP50* or *PRMT5* mRNA (median cutoff setting) was determined from the online tool (https://www.kmplot.com). 

The survival curves for each breast cancer subgroup were obtained using the PAM50 setting on the website (Basal for TNBC, HER2, luminal B and Luminal A). The table below indicates the number of patients retrieved with the MEP50 and PRMT5 probe sets within each breast cancer subgroup (Table 3).

The survival curves for each TNBC subtype were retrieved using the Pietenpol setting classifying the TNBC subtypes as reported by the group of Prof. Pietenpol [2] (basal-like 1, BL1; basal-like 2, BL2; immunomodulatory, IM; mesenchymal, Mes; mesenchymal stem-like, MSL; luminal androgen receptor, LAR). The table below indicates the number of patients retrieved with the MEP50 and PRMT5 probe sets within each TNBC subtype (Table 4).

The obtained Hazard Ratio (HR) with 95% confidence interval and log-rank *p*-values were generated automatically from the online tool (https://www.kmplot.com) and are shown on the corresponding Figures.

### 2.3. Cell Culture

HCC38, MDA-MB-231 and MDA-MB-453 TNBC cells were purchased from the American Type Culture Collection (ATCC, LGC Promochem, Karnataka, India), authenticated by short tandem repeat profiling in 2021 (not shown). HCC38 cells were cultured in RPMI-1640 (LifeTechnologies, Carlsbad, CA, USA) supplemented with 10% (*vol/vol*) fetal bovine serum (FBS, LifeTechnologies), 1.5 g/L sodium bicarbonate (LifeTechnologies), 10 mmol/L Hepes (LifeTechnologies), 1 mmol/L sodium pyruvate (LifeTechnologies), 100 U/mL penicillin, and 100 µg/mL streptomycin (LifeTechnologies). MDA-MB-453 and MDA-MB-231 cells were cultured in DMEM-F12 (LifeTechnologies) supplemented with 10% FBS, 100 U/mL penicillin, and 100 µg/mL streptomycin.

### 2.4. Validation of the MEP50 Antibodies for Immunohistochemistry (IHC) Staining

HCC38 and MDA-MB-231 cells were transfected with 20 nM of control (Allstars negative control, ref: SI03650318, Qiagen, Hilden, Germany) or MEP50 (ref: SI03152730, Qiagen, target sequence 5′-ATGCTAGATCTGTGCCGTTAA-3′) siRNA using INTERFERin (Polyplus Transfection, Illkirch-Graffenstaden, France). Forty-eight hours post transfection, protein lysates were extracted from one plate and subjected to Western blot, as previously described [48,52,53], to confirm MEP50 depletion efficiency. The other plates were used to validate the specificity of the MEP50 antibodies for IHC purpose: about 10 million cells per condition (control or MEP50 siRNA treated cells) were pelleted, then fixed with the same fixator (AFA: Alcohol, Formalin, Acetic acid) used to fix the human samples. Fixed cells were then paraffin embedded, and 3 µm-thick sections were cut with a microtome and then stained as the human samples of the Curie cohort.

### 2.5. Validation of the H4R3me2s Antibodies for IHC Staining

MDA-MB-453 cells were incubated for 48 h with vehicle (DMSO) or 1 µM of EPZ015666 (PRMT5 inhibitor, Clinisciences, Nanterre, France). Protein lysates were extracted from one plate, and Western blot analysis was performed using a pan symmetric dimethyl-arginine (SDMA) antibody to confirm the efficacy of EPZ015666. The other plates were used to validate the specificity of the H4R3me2s antibodies for IHC purpose: about 10 million cells per condition (DMSO or EPZ015666 treated cells) were pelleted, then fixed with the same fixator (AFA) used to fix the human samples. Fixed cells were then paraffin-embedded, and 3 µm-thick sections were cut with a microtome, and stained as the human samples of the Curie cohort.

### 2.6. Immunohistochemistry on Human Samples

IHC was performed on the following number of tumors of our Curie cohort (TNBC: *n* = 26; HER2: *n* = 26; luminal A: *n* = 17; luminal B: *n* = 25) and normal breast tissues (*n* = 7) (Table 5).

AFA-fixed paraffin-embedded tissues, obtained at the time of the initial diagnosis, were retrieved from the archives of the Department of Pathology of Institut Curie Hospital. Three µm-thick sections were cut with a microtome from the paraffin-embedded tissue blocks, and tissue microarrays (TMA) were made. Tissue sections were dewaxed and rehydrated through a series of xylene and ethanol washes before heat-induced epitope retrieval. Antigen retrieval was performed in EDTA buffer pH = 6 (MEP50) or pH = 9 (H4R3me2s). The slides were incubated with primary antibodies against MEP50 (1/1000, 1 h at room temperature) or H4R3me2s (1/1000, 15 min at room temperature). Then, the slides were incubated with secondary antibodies coupled to horseradish peroxidase. A DAB (3,3′-Diaminobenzidine) solution was applied for 5 min for revelation of peroxidase. Slides were counterstained with hematoxylin before mounting with resin. Immunostaining was processed by using a Dako automated machine.

For surface staining quantifications, whole digital slide images were obtained using virtual microscopy (Philips Ultra-Fast Scanner 1.6 RA, Amsterdam, The Netherlands) and analyzed with Digital Image Analysis platform HALO (version 3.0.311.218; Indica Lab, Albuquerque, NM, USA). Tissue classifier was trained to segment the tissue image into tumor (epithelial cells) or stromal compartment. Area Quantification module (v2.1.3, Albuquerque, NM, USA) was used to evaluate the total area of epithelial compartment and the area of tissue positive for MEP50/H4R3me2s staining.

For subcellular localization, MEP50 staining was studied at the nuclear, plasma membrane and cytoplasmic compartments by two pathologists (A.E. and D.M., coauthors of this article) from the Institut Curie Hospital. For each tumor sample, the pathologists assigned IHC scores for MEP50/H4R3me2s staining based on the proportion of positive cells and its corresponding immunostaining intensity for each cellular compartment (only nuclear for H4R3me2s) by the following formula:

*IHC score = percentage of stained cells x intensity of immunostaining*

Hence, each score ranged between 0 and 3 (0: no staining; 3: strongest staining).

### 2.7. Antibodies

The primary antibodies used for Western blotting were: MEP50 (Cell Signaling Technology, ref. #2018, Danvers, MA, USA), PRMT5 (Merck Millipore, ref. #07-405, Burlington, MA, USA), pan symmetric dimethyl-arginine (SDMA) antibody (Cell Signaling Technology, ref. #13222), β-actin (Sigma-Aldrich, Ref. #A5441, St. Louis, MI, USA), and GAPDH (Cell Signaling Technology, ref. #2118). The primary antibodies used for IHC were: MEP50 (Cell Signaling Technology, ref. #2018) and H4R3me2s (Abcam, Ref. #ab5823, Cambridge, UK).

### 2.8. Statistical Analysis

R software and GraphPad Prism 9 were used for statistical analyses. Pearson correlation was used to estimate an association between two variables. An ANOVA test was used to calculate the *p*-values when comparing the expression of MEP50/H4R3me2s between two different breast cancer groups. 

## 3. Results

### 3.1. TNBC Express Higher Levels of MEP50 mRNA Compared to the Other Breast Cancer Subgroups and Normal Breast Tissues

**Figure 1:**
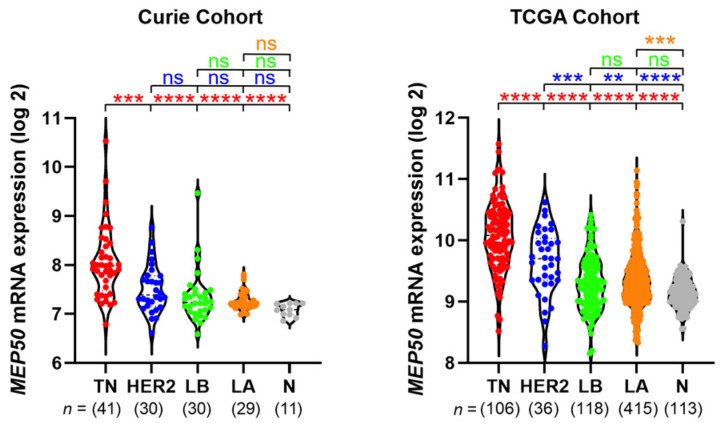
TNBC express high levels of *MEP50* mRNA. *MEP50* mRNA expression in the different breast cancer subgroups and in normal breast tissues in Curie (**left panel**) and TCGA (**right panel**) cohorts. The breast cancer subgroups are ordered left to right from the most to the least proliferative tumors: TNBC (TN, red), HER2 (blue), luminal B (LB, green), luminal A (LA, orange). Normal breast tissues (N) are in grey. Relative RNA quantifications are logarithmically (log2) transformed and illustrated by violin plots with each sample represented by a circle. The statistics in red indicate the comparison vs. TN, in blue vs. HER2, in green vs. LB, and in orange vs. LA: ns (not significant), ** *p* < 0.01, *** *p* < 0.001, **** *p* < 0.0001.

At the RNA level, *MEP50* has been reported to be overexpressed in breast cancer compared to the normal breast tissue, without accounting for breast cancer heterogeneity [28,29,30]. Here, we examined *MEP50* mRNA expression in the different breast cancer subgroups. *MEP50* mRNA is heterogeneously expressed within each subgroup, particularly in TNBC, with some tumors expressing low and others high levels of *MEP50* mRNA. Nevertheless, we observed higher levels of *MEP50* mRNA in TNBC compared to the other breast cancer subgroups and normal tissues in both Curie (Table 1) and TCGA (Table 2) cohorts (Figure 1). We observed a positive correlation between *MEP50* and *PRMT5* mRNA levels in our cohort in the whole breast cancer population but not within the different breast cancer subgroups, although a tendency was observed for the luminal B subgroup (*p* = 0.053) (Figure S1).

### 3.2. MEP50 and PRMT5 mRNA Levels Associate with Recurrence-Free Survival in TNBC and Luminal B Breast Tumors

**Figure 2:**
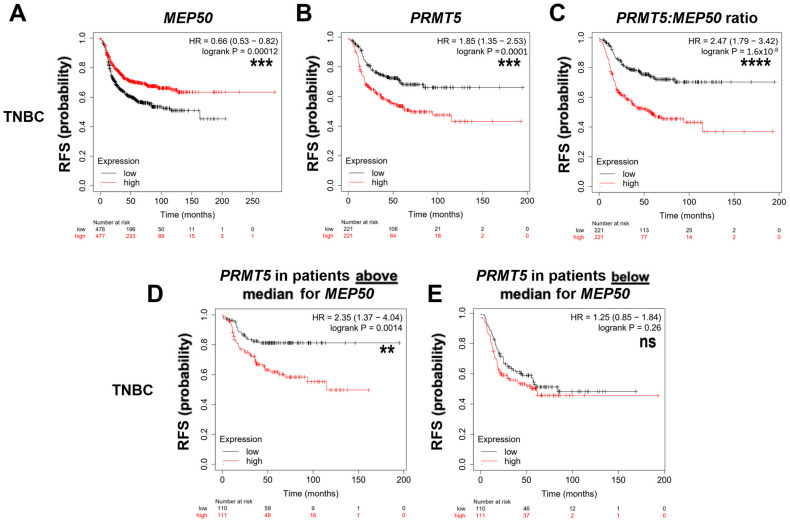

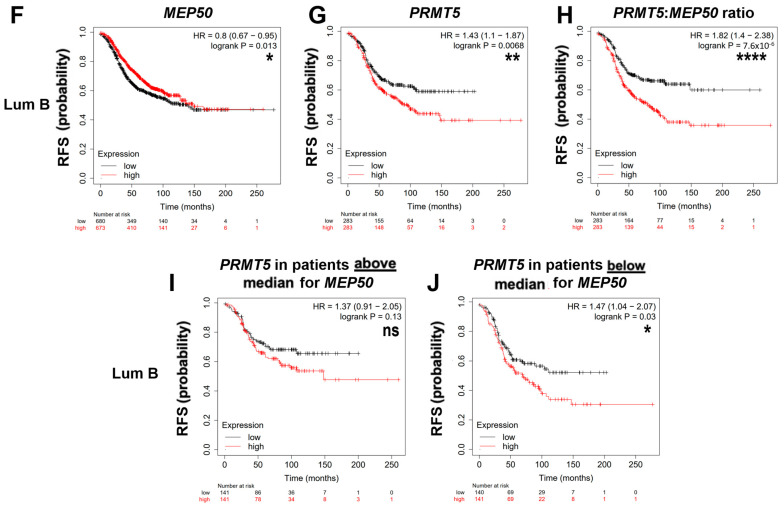
High *MEP50* mRNA expression is associated with a better prognosis in TNBC and luminal B subgroups. (**A**–**C**,**F**–**H**). Recurrence-free survival (RFS) based on *MEP50* or *PRMT5* mRNA expression or *PRMT5:MEP50* mRNA ratio were obtained from the Kaplan–Meier (KM) plotter website (http://kmplot.com) for TNBC (**A**–**C**) and luminal B (Lum B; (**F**–**H**)). (**D**,**E**,**I**,**J**). RFS based on *PRMT5* mRNA expression within patients having either high (above median, (**D**,**I**)) or low (below median, (**E**,**J**)) *MEP50* mRNA expression (median cutoff). Of note, more patients were retrieved with MEP50 probe set compared to the PRMT5 probe set (Table 3). The obtained Hazard Ratio (HR) with 95% confidence interval and log-rank *p*-values are shown. ns (not significant), * *p* < 0.05, ** *p* < 0.01, *** *p* < 0.001, **** *p* < 0.0001.

We examined whether the expression of *MEP50* mRNA was linked to prognosis on the Kaplan–Meier (KM) plotter online database (www.kmplot.com) [51]. High *MEP50* mRNA levels were associated with a more favorable RFS in TNBC (*p* = 1.2 × 10^−4^) and luminal B (*p* = 0.013) tumors (Figure 2A,F). In contrast, high *PRMT5* mRNA levels were associated with worse RFS in these two subgroups (TNBC, *p* = 1.0 × 10^−4^; luminal B, *p* = 6.8 × 10^−3^; Figure 2B,G). Then, we sought to examine if the tumors with worse RFS were those expressing both high *PRMT5* and low *MEP50* mRNA. For this purpose, we analyzed the prognostic value of *PRMT5 or MEP50* mRNA in the two subpopulations expressing high (> median) or low (< median) *MEP50 or PRMT5* mRNA levels in the different breast cancer subgroups.

In TNBC patients with high *MEP50* (red line with good prognosis, Figure 2A), additionally considering high *PRMT5* expression unveils a population associated with poor prognosis (Figure 2D, *p* = 0.001). However, *PRMT5* expression showed no added prognostic value (Figure 2E, *p* = 0.26) in the TNBC population that expressed low *MEP50* (already associated with a poor prognosis, black line Figure 2A). Conversely, considering low *MEP50* expression within the group of patients with either high (red line Figure 2B) or low (black line Figure 2B) *PRMT5* reveals a population of patients with a worse prognosis (Figure S2A, *p* = 0.012; Figure S2B, *p* = 7.7 ×10^−5^). These observations indicate that considering both *MEP50* and *PRMT5* mRNA levels, instead of each separately, aids in improving patient stratification for RFS. Accordingly, the *PRMT5*:*MEP50* mRNA ratio is more significantly associated with RFS (*p* = 1.6 × 10^−8^; Figure 2C) than *PRMT5* alone (*p* = 1.0 × 10^−4^; Figure 2B).

In luminal B patients with high (red line in Figure 2F) or low (black line in Figure 2F) *MEP50*, considering high *PRMT5* expression unveils a population associated with poor prognosis (Figure 2I,J). However, *PRMT5* expression added a significant prognostic value only in luminal B expressing low levels of *MEP50* mRNA (although associated with poor prognosis, Figure 2J, *p* = 0.03). As for TNBC, a high *PRMT5*:*MEP50* mRNA ratio is more significantly associated with a poor prognosis (*p* = 7.6 × 10^−6^; Figure 2H) than high *PRMT5* alone (*p* = 6.8 × 10^−3^; Figure 2G).

In contrast to TNBC and luminal B, *MEP50*, *PRMT5,* and *PRMT5:MEP50* mRNA levels were not associated with RFS in luminal A nor HER2 subgroups (Figure S3). 

Together, these observations indicate that *PRMT5* and *MEP50* mRNA levels are inversely associated with prognosis in TNBC and luminal B breast tumors. Taking into account both *PRMT5* and *MEP50* mRNA levels helps to better stratify patients associated with poor prognosis in these two breast cancer subgroups. This suggests that TNBC or luminal B tumors harboring high *PRMT5* and/or low *MEP50* mRNA levels could be at a higher risk of recurrence. 

### 3.3. MEP50 and PRMT5 mRNA Levels Are Associated with Prognosis in Some TNBC Subtypes

**Figure 3:**
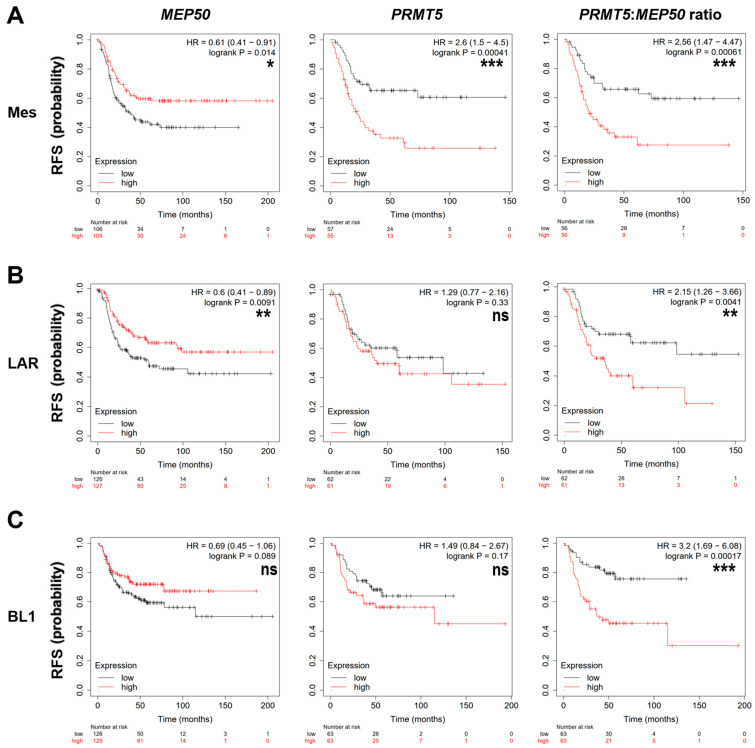
High *PRMT5:MEP50* mRNA ratio is associated with worse RFS in mesenchymal, LAR and BL1 TNBC subtypes. (**A**–**C**). RFS based on *MEP50* or *PRMT5* mRNA expression or *PRMT5:MEP50* mRNA ratio were obtained from the Kaplan–Meier (KM) plotter website (http://kmplot.com) for mesenchymal (Mes; (**A**)), luminal androgen receptor (LAR; (**B**)), and basal-like 1 (BL1; (**C**)) TNBC subtypes. Of note, more patients were retrieved with MEP50 probe set compared to the PRMT5 probe set (Table 4). The obtained Hazard Ratio (HR) with 95% confidence interval and log-rank *p*-values are shown. ns (not significant), * *p* < 0.05, ** *p* < 0.01, *** *p* < 0.001.

As we observed that *MEP50* mRNA level is significantly associated with RFS in TNBC, we analyzed whether it is the case in the different TNBC subtypes using the KM-plotter database (www.kmplot.com) [51]. Despite smaller sample sizes (Table 4), high levels of *MEP50* mRNA were associated with better RFS in the mesenchymal (*p* = 0.014) and LAR (*p* = 0.009) subtypes (Figure 3A,B, left panels). A similar trend was observed in BL1 but was not statistically significant (*p* = 0.089; Figure 3C, left panel). 

Strikingly, high *PRMT5* mRNA levels were associated with worse RFS only in the mesenchymal (*p* = 4.1 × 10^−4^; Figure 3A, middle panel) and not in the other TNBC subtypes (Figure 3B,C, middle panel, Supplementary Figure S4, middle panels).

The *PRMT5*:*MEP50* ratio significantly improved the prognostic value in the LAR (*p* = 0.004) and BL1 (*p* = 1.7 × 10^−4^) subtypes (Figure 3B,C, right panels) than *PRMT5* or *MEP50* alone. This was not the case for the mesenchymal subtype (Figure 3A, right panel) in which *PRMT5* alone was already significantly highly associated with a bad prognosis (Figure 3A, middle panel). 

*MEP50*, *PRMT5,* and *PRMT5:MEP50* mRNA levels were not associated with RFS in the BL2, IM, and MSL TNBC subtypes (Figure S4).

### 3.4. MEP50 Exhibits Differential Subcellular Localization in Breast Cancer Compared to Normal Breast Tissues

**Figure 4:**
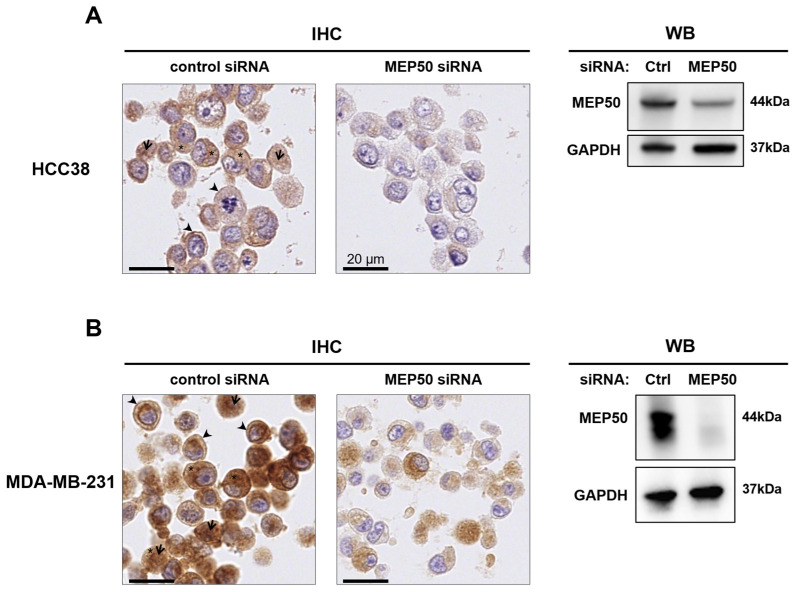
Validation of a MEP50 antibody suitable for IHC staining. HCC38 (**A**) and MDA-MB-231 (**B**) cells were treated with 20 nM control (Ctrl) siRNA or siRNA targeting MEP50 for 48 h. Cells were pelleted, fixed with AFA, paraffin embedded, and subjected to IHC staining with an anti-MEP50 antibody (scale bars = 20 μm) ((**A**,**B**), left panels). MEP50 depletion was verified by Western blotting (WB) using an anti-MEP50 antibody ((**A**,**B**), right panels). Anti-GAPDH antibodies were used as a loading control. Arrows, arrowheads, and asterisks indicate MEP50 staining in the nucleus, plasma membrane, and cytoplasm, respectively. The uncropped blots are shown in Figure S5.

Next, we examined MEP50 expression at the protein level in the different breast cancer subgroups of the Curie cohort. We first validated an anti-MEP50 antibody for IHC purposes by staining two TNBC cell lines (HCC38 and MDA-MB-231) depleted or not for MEP50 using MEP50 siRNA and fixed using the same protocol as the one used for fixing the human tissues (Figure 4). IHC staining revealed that MEP50 was mainly expressed in the cytosol in both cell lines but was also detected at the plasma membrane and in the nucleus in some cells (Figure 4, left panels). The IHC staining decreased/disappeared in MEP50-depleted cells, demonstrating the specificity of the antibody (Figure 4, left panels). Western blot analysis confirmed the depletion of MEP50 in cells treated with MEP50 siRNA (Figure 4, right panels).

**Figure 5:**
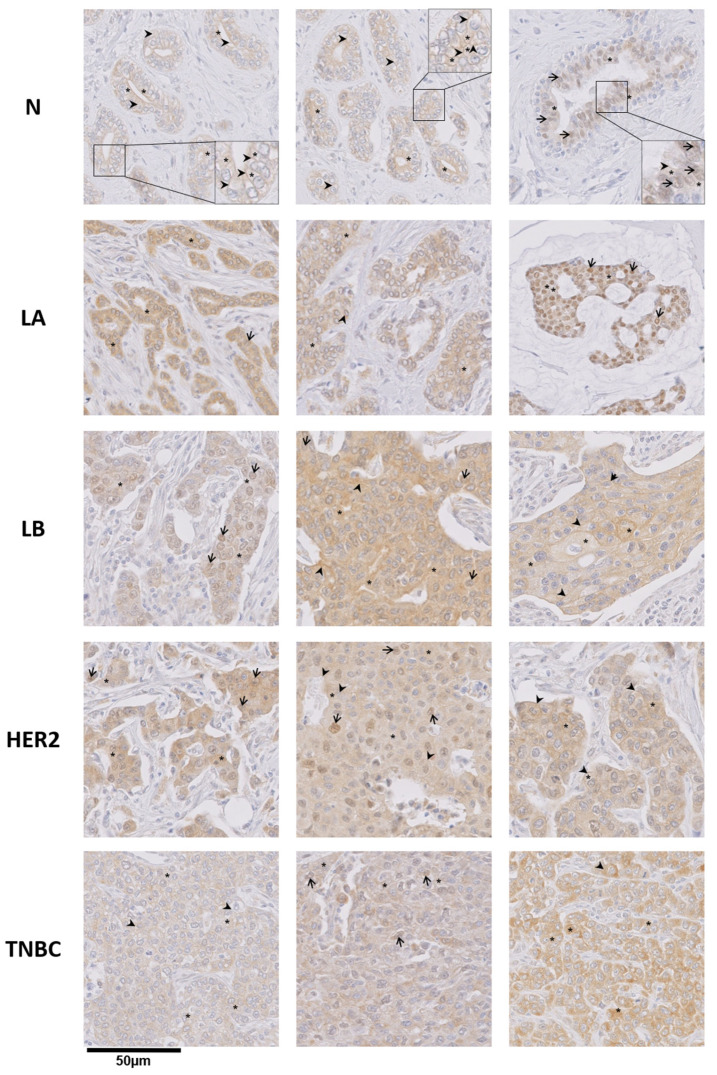
MEP50 is more expressed in breast cancer subgroups compared to normal breast tissues. The expression and localization of MEP50 protein were analyzed by IHC in the Curie cohort. Three representative images of MEP50 staining are shown for the different breast cancer subgroups and normal breast tissue to illustrate its heterogeneous expression and distribution (scale bar = 50 μm). To better visualize cytoplasmic MEP50 and plasma membrane-associated MEP50 in normal samples, a part of the image is shown with a higher magnification (2×) in the inset. Arrows, arrowheads, and asterisks indicate MEP50 staining in the nucleus, plasma membrane, and cytoplasm, respectively.

**Figure 6:**
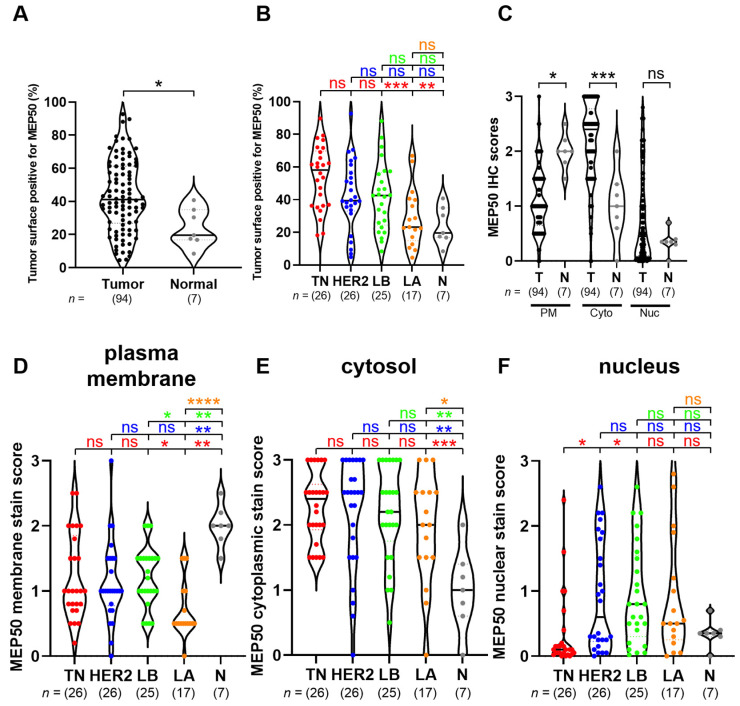
Differential subcellular localization of MEP50 among the breast tumor samples and normal breast tissues. Higher percentage of MEP50-expressing tumor cells compared to the normal breast tissues (**A**). Higher percentage of MEP50-expressing tumor cells in TNBC compared to the other breast cancer subgroups and normal breast tissues (**B**). Quantification of the tumoral surface positive for MEP50 staining represented as a percentage compared to the total epithelial surface (**A**,**B**). MEP50 staining was scored at the plasma membrane (**C**,**D**), in the cytosol (**C**,**E**) and in the nucleus (**C**,**F**) in the samples of the Curie cohort (from Figure 5). The score was obtained by combining the percentage and the intensity of the staining of the epithelial cells (0: no staining, 3: the strongest staining). Tumor (T, black), TNBC (TN, red), HER2 (blue), luminal B (LB, green), luminal A (LA, orange), and normal breast tissues (N, grey). PM: plasma membrane; Cyto: cytosol; Nuc: nucleus. The statistics in red indicate the comparison vs. TN, in blue vs. HER2, in green vs. LB, and in orange vs. LA: ns (not significant); * *p* < 0.05; ** *p* < 0.01; *** *p* < 0.001; **** *p* < 0.0001.

Using the validated MEP50 antibody for IHC purpose, we next analyzed MEP50 expression in breast cancer samples and normal breast tissues of our Curie cohort (Figure 5). MEP50 was more expressed in breast cancer compared to normal breast tissues (*p* = 0.02) (Figure 5 and Figure 6A). Moreover, TNBC had higher MEP50-expressing tumor cells compared to luminal A and normal breast tissues (Figure 6B). MEP50 was detected in the cytosol (Figure 5, asterisks), in the nucleus (Figure 5, arrow), and also at the plasma membrane of some cells (Figure 5, arrowhead). To quantify the subcellular localization of MEP50, we scored its staining at the plasma membrane (Figure 6C,D), in the cytosol (Figure 6C,E) and in the nucleus (Figure 6C,F) of the different breast cancer subgroups and in normal breast tissues. We observed a high heterogeneity of the MEP50 score, either at the plasma membrane, in the cytosol or in the nucleus, within each analyzed group (Figure 6D–F). Nevertheless, breast tumors had significantly lower levels of MEP50 at the plasma membrane (Figure 6C,D) but higher levels of cytoplasmic MEP50 (Figure 6C,E) compared to normal breast tissues. In the nucleus, there was no significant difference between breast tumors and normal breast tissues (Figure 6C). However, the TNBC subgroup had the lowest MEP50 expression compared to the other groups and normal tissue, but this was only significant with luminal B and HER2 subgroups (Figure 6F).

Altogether, our study highlights the differential subcellular localization of MEP50 between cancerous and normal breast tissues. Importantly, its subcellular distribution is highly heterogenous within the cancer tissues as well as normal breast tissues. 

### 3.5. The Most Aggressive Breast Cancer Subgroups Display the Lowest Levels of PRMT5-Dependent Symmetric Dimethylation of Histone H4 (H4R3me2s)

**Figure 7:**
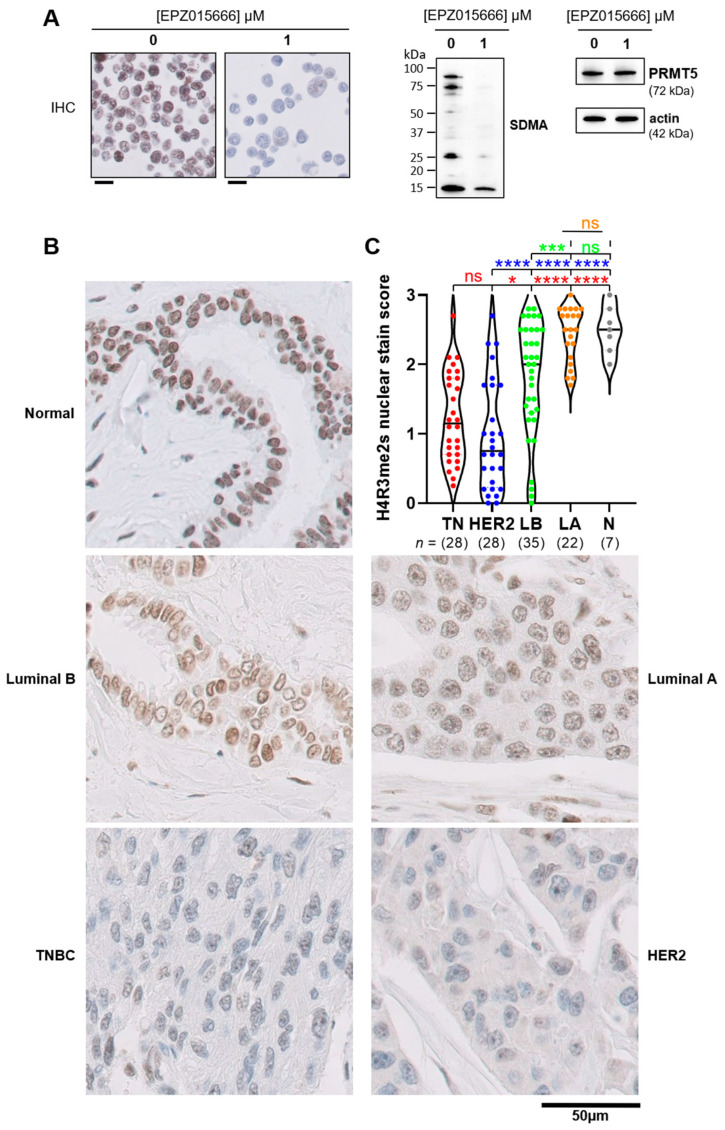
The hormone receptor-negative breast tumors express lower levels of symmetrically dimethylated histone H4 on Arginine 3 (H4R3me2s) compared to the other breast cancer subgroups and normal breast tissues. (**A**). Validation of an H4R3me2s antibody suitable for IHC staining. MDA-MB-453 cells were incubated with 1 µM of a PRMT5 inhibitor (EPZ015666) or with DMSO as a control for 48 h. Cells were pelleted, fixed with AFA, paraffin-embedded and subjected to IHC staining with an anti-H4R3me2s antibody (scale bars = 20 μm) (left panel). PRMT5 inhibition was verified by Western blotting using an anti-pan symmetric dimethyl-arginine (SDMA) antibody, and anti-PRMT5 and anti-actin antibodies were used as loading controls (right panel). The uncropped blots are shown in Figure S5. (**B**). Histone H4 is highly symmetrically dimethylated on arginine 3 in normal breast tissues. The symmetric dimethylation of H4R3 was analyzed by IHC in the Curie cohort. A representative image of H4R3me2s staining is shown for the different breast cancer subgroups and normal breast tissue (scale bar = 50 μm). (**C**). Hormone negative breast tumors (TNBC and HER2) display low levels of H4R3me2s. Nuclear H4R3me2s staining was scored by combining the percentage and the intensity of the staining of the epithelial cells (0: no staining, 3: the strongest staining). TNBC (TN, red), HER2 (blue), luminal B (LB, green), luminal A (LA, orange), and normal breast tissues (N, grey). The statistics in red indicate the comparison vs. TN, in blue vs. HER2, in green vs. LB, and in orange vs. LA: ns, (not significant); * *p* < 0.05, *** *p* < 0.001, **** *p* < 0.0001.

Next, we sought to determine whether the low nuclear expression of MEP50 (this study) and PRMT5 [14] observed in TNBC correlated with low nuclear PRMT5 activity. We measured the level of histone H4 symmetrically dimethylated on Arginine 3 (H4R3me2s) as a marker of PRMT5 nuclear activity. First, we validated an antibody targeting H4R3me2s for IHC purposes in a TNBC cell line (MDA-MB-453) treated with a PRMT5 inhibitor (EPZ015666) and fixed using the same conditions as the human samples of our Curie cohort (Figure 7A). PRMT5 inhibition reduced the methylation of histone H4 (H4R3me2s) as observed by IHC (Figure 7A, left panel), validating this antibody. Western blot analysis confirmed that EPZ015666 lowered PRMT5 activity, using a pan antibody detecting symmetric dimethylated arginine (SDMA) (Figure 7A, right panel). 

Normal breast tissues and luminal A breast cancer displayed the highest scores for H4R3me2s compared to the other breast cancer tissues (Figure 7B,C). The hormone-negative tumors (TNBC and HER2) had similar scores for H4R3me2s staining which was the least compared to the other groups (Figure 7B,C). The highly heterogenous H4R3me2s staining score is noteworthy within each analyzed group, particularly in TNBC, HER2, and luminal B subgroups (Figure 7C).

Together, our findings indicate that histone H4 is highly symmetrically dimethylated on arginine 3 in normal breast tissue. Low H4R3 dimethylation appears to be associated with the most proliferative breast cancer subgroups (TNBC and HER2). 

## 4. Discussion

*MEP50* mRNA has previously been shown to be overexpressed in breast cancer [28,29,30], but no study has explored its expression within the different breast cancer subgroups. In this study, we report that the highest expression of *MEP50* mRNA is found in TNBC when compared to luminal A, luminal B, and HER2 breast cancer subgroups, and to normal breast tissues. 

Previous studies have shown that high *MEP50* [35] and high *PRMT5* [54] mRNA levels are associated with worse prognosis in the whole breast cancer population. However, as the different breast cancer subgroups are associated with different prognosis, it is crucial to perform survival analyses within each subgroup and not in the entire breast cancer population. Due to too few clinical events, we were not able to analyze the correlation between *MEP50* mRNA expression and survival in our cohort. Nevertheless, using the KM plotter database (www.kmplot.com) [51], we find that high *MEP50* mRNA is associated with better RFS in TNBC and luminal B patients, with the highest statistical significance in TNBC. Using the same website, Liu and colleagues have reported that high *MEP50* mRNA is associated with a worse prognosis in the entire breast tumor population [35]. In contrast, we observed that high *MEP50* mRNA is associated with better RFS in whole breast cancer patients (*p*-value = 2.9 × 10^−4^; 201421_s_at probe set; Figure S6). Like *MEP50*, we found that *PRMT5* mRNA is associated with RFS only in TNBC and luminal B patients and not in the other breast cancer subgroups. This is in agreement with previous reports showing that high *PRMT5* mRNA levels are associated with worst overall survival and distant metastasis-free survival in TNBC [14,55,56]. Unexpectedly, *MEP50* and *PRMT5* mRNA levels correlate with RFS in an inverse manner, with high PRMT5-expressing patients harboring worse prognosis. This observation suggests that MEP50 and PRMT5 could have specific, independent functions, and not always work together within their well-described hetero-octameric protein complex. However, here the prognosis analysis was performed with the mRNA and not protein expression, and thus, it is also possible that *PRMT5* and *MEP50* are differentially regulated at the post-transcriptional level. Interestingly, considering the expression of both *MEP50* and *PRMT5* mRNA further stratifies patients according to their survival outcome. The *PRMT5*:*MEP50* mRNA ratio could therefore be a valuable prognostic marker to predict RFS in TNBC and luminal B patients. 

TNBC itself is highly heterogenous, with each TNBC subtype being associated with a different prognosis. Among the different TNBC subtypes, the mesenchymal and LAR subtypes have the highest residual cancer burden following neoadjuvant chemotherapy [57]. Low *MEP50* mRNA is associated with worse RFS only in mesenchymal and LAR, the most chemo-resistant TNBC subtypes, suggesting that mesenchymal and LAR TNBC patients expressing low *MEP50* mRNA, could be more prone to recurrence after chemotherapy. Strikingly, high *PRMT5* mRNA is strongly associated with poor RFS in the mesenchymal subtype, which is enriched in the epithelial-to-mesenchymal transition (EMT) pathway [2,3,4]. The EMT pathway is implicated in invasion, tumor dissemination and drug resistance [58,59,60,61]. As PRMT5 depletion or inhibition impairs EMT [17,28,62,63,64], mesenchymal TNBC may represent a niche for PRMT5 inhibitors. When considering the *PRMT5*:*MEP50* mRNA expression ratio, a significant correlation is observed with poorer RFS in three subtypes: mesenchymal, LAR, and BL1. The efficacy of PRMT5 inhibitors, alone or in combination with other drugs, in luminal B and certain TNBC subtypes with high *PRMT5*:*MEP50* ratio needs to be explored.

Similar to the RNA levels, TNBC express higher protein level of MEP50 compared to normal breast tissues. MEP50 protein localizes to the cytosol, nucleus and the plasma membrane with some notable differences between breast tumors and normal breast tissues. Normal breast tissues express higher MEP50 (in this study) and PRMT5 [14] compared to breast cancer at the plasma membrane. Therefore, in normal breast tissues, PRMT5 and MEP50 may form a complex at the plasma membrane whose physiological relevance is yet to be discovered. Breast cancer tissues express more cytosolic MEP50 than normal breast tissue. Cytoplasmic MEP50 is linked to proliferation in prostate cancer cells, whereas nuclear MEP50 is associated with differentiation [37,38,39,40,41,42]. However, in our study, cytoplasmic MEP50 appears to be a marker of breast cancer rather than being linked to proliferation, since luminal A tumors are poorly proliferative but still display high cytosolic MEP50. This indicates that cytoplasmic MEP50 localization is cancer-type specific (between the different breast cancer subgroups and between breast and prostate cancers). TNBC express the least nuclear MEP50 but it is significant only when compared to luminal tumors. 

We have previously shown that TNBC express low levels of nuclear PRMT5 compared to the other breast cancer subgroups and to normal tissues [14], suggesting a lower nuclear PRMT5 activity in TNBC. Symmetric dimethylation of H4R3 is one of the readouts for PRMT5 nuclear activity. We observe the highest H4R3me2s staining in the normal breast tissue and luminal A subgroup and the lowest in the most proliferative breast cancer subgroups (TNBC and HER2). Thus, high H4R3me2s is associated with good prognosis in breast cancer. This PRMT5-dependent histone mark is a transcriptional repressor, suggesting that it may repress a subset of genes linked to proliferation and survival of cancer cells. Further exploring the methylation status of cytosolic and other nuclear PRMT5 substrates may help us to better understand the implication of PRMT5 in breast cancer. 

## 5. Conclusions

This comprehensive study explores the RNA and protein expression of the main PRMT5 protein partner, MEP50, in the different breast cancer subgroups. High *MEP50* is found in TNBC and is associated with a better RFS in the whole TNBC population and in the LAR and mesenchymal TNBC subtypes. Distinct subcellular localization of MEP50 is a potential marker of breast cancer. The PRMT5-mediated methylation of histone H4 (H4R3me2s), which is low in TNBC and HER2, is linked with the good prognosis-associated luminal A tumors. 

## Figures and Tables

**Table 1:** Curie Cohort (Transcriptome Analysis): *MEP50* mRNA Expression in Breast Cancer Subgroups and Normal Breast Tissues (Figure 1).

Number of Samples in Breast Cancer Subgroups and in Normal Breast Tissues				
TNBC	HER2	Luminal B	Luminal A	normal breast tissues
41	30	30	29	11

**Table 2:** TCGA cohort (transcriptome analysis): *MEP50* mRNA expression in breast cancer subgroups and normal breast tissues (Figure 1).

Number of Samples in Breast Cancer Subgroups and in Normal Breast Tissues				
TNBC	HER2	Luminal B	Luminal A	normal breast tissues
106	36	118	415	113

**Table 3:** Survival analyses in the breast cancer subgroups (Figure 2).

	Number of Patients Retrieved on https://www.kmplot.com with the MEP50 (201421_s_at) or the PRMT5 (1564520_s_at) Probe Sets			
	TNBC	HER2	Luminal B	Luminal A
*MEP50* mRNA	953	695	1353	1809
*PRMT5* mRNA	442	358	566	631
PRMT5:MEP50 mRNA	442	358	566	631

**Table 4:** Survival analyses in TNBC subtypes (Figure 3).

	Number of Patients Retrieved on https://www.kmplot.com with the MEP50 (201421_s_at) or the PRMT5 (1564520_s_at) Probe Sets					
	BL1	BL2	IM	Mes	MSL	LAR
*MEP50* mRNA	251	101	300	211	81	253
*PRMT5* mRNA	126	68	130	112	43	123
PRMT5:MEP50 mRNA	126	68	130	112	43	123

**Table 5:** Curie cohort (IHC): MEP50 expression (MEP50 staining, Figures 5 and 6) and PRMT5 activity (H4R3me2s staining, Figure 7) in breast cancer subgroups and normal breast tissues.

Number of Samples in Breast Cancer Subgroups and in Normal Breast Tissues				
TNBC	HER2	Luminal B	Luminal A	normal breast tissues
26	26	25	17	7

## Data Availability

The data presented in this study are available in this article (and Supplementary Materials).

## Supplementary Materials

The following are available online at https://www.mdpi.com/article/10.3390/cancers14194766/s1, Figure S1: Correlation analyses between *MEP50* and *PRMT5* mRNA expression in the Curie cohort, Figure S2: RFS based on *MEP50* mRNA expression levels within high (above median, left panel) or low (below median, right panel) *PRMT5* mRNA expression (median cutoff) in the different breast cancer subgroups, Figure S3: *MEP50* mRNA expression levels are not associated with RFS in HER2 and luminal A breast cancers, Figure S4: *PRMT5*:*MEP50* mRNA ratio is not associated with RFS in BL2, IM and MSL TNBC subtypes. Figure S5: Uncropped membranes of Western blots corresponding to Figure 4A (A) and Figure 7A (B). Figure S6: High MEP50 mRNA expression is associated with a better prognosis in all breast cancers.
