# Supplementary material for: Expression, Localization and Prognosis Association of MEP50 in Breast Cancer
Source: Cancers (Basel). 2022 Sep 29;14(19):4766. doi: 10.3390/cancers14194766 (PMC9563057; doi:10.3390/cancers14194766)
Supplement: Supplementary file 1 [file cancers-14-04766-s001.zip › cancers-1923618-supplementary.pdf]

Supplementary information for

Expression, localization and prognosis-association of MEP50 in breast cancer

Samyuktha Suresh, Mathilde Vinet, Rayan Dakroub, Laetitia Lesage, Mengliang Ye, Hussein Fayyad-Kazan, André Nicolas, Didier Meseure, and Thierry Dubois
Correspondence to: thierry.dubois@curie.fr

This PDF file includes:

Supplementary Figures S1 to S6

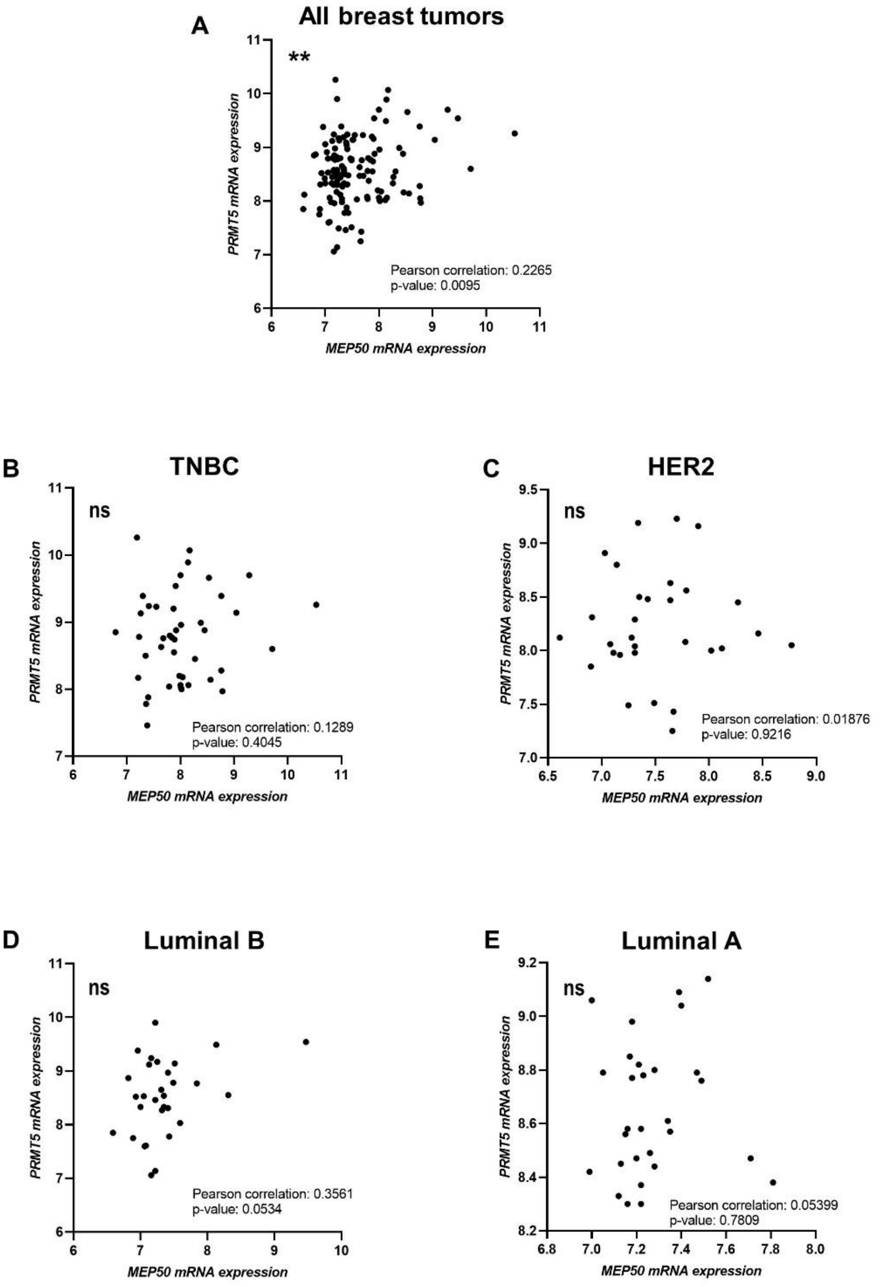

Supplementary Figure S1. Correlation analyses between *MEP50* and *PRMT5* mRNA expression in the Curie cohort. Pearson correlation between the *MEP50* and *PRMT5* mRNA was performed in the curie cohort in the whole breast cancer population (A), TNBC (B), HER2 (C), luminal B (D), and luminal A (E). ns (not significant), ** p<0.01.

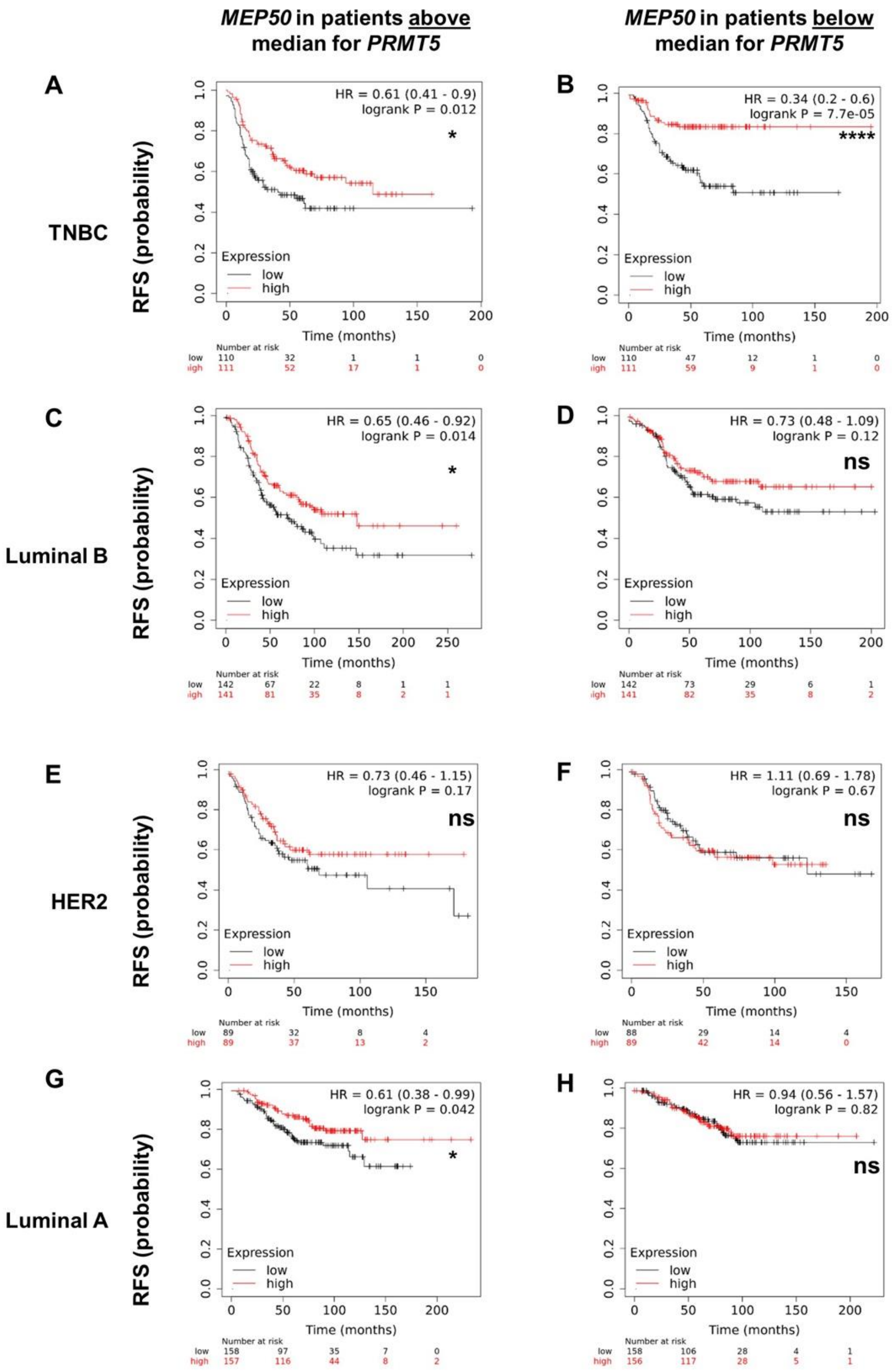
MEP50 in patients above median for PRMT5
MEP50 in patients below median for PRMT5
A
B
C
D
E
F
G
H
TNBC
Luminal B
HER2
Luminal A
RFS (probability)
Time (months)
Expression
low
high
Number at risk
HR = 0.61 (0.41 - 0.9)
logrank P = 0.012
*
HR = 0.34 (0.2 - 0.6)
logrank P = 7.7e-05
****
HR = 0.65 (0.46 - 0.92)
logrank P = 0.014
*
HR = 0.73 (0.48 - 1.09)
logrank P = 0.12
ns
HR = 0.73 (0.46 - 1.15)
logrank P = 0.17
ns
HR = 1.11 (0.69 - 1.78)
logrank P = 0.67
ns
HR = 0.61 (0.38 - 0.99)
logrank P = 0.042
*
HR = 0.94 (0.56 - 1.57)
logrank P = 0.82
ns

Figure S2

Supplemental Figure S2. RFS based on *MEP50* mRNA expression levels within high (above median, left panel) or low (below median, right panel) *PRMT5* mRNA expression (median cutoff) in the different breast cancer subgroups. Data were obtained from the Kaplan-Meier (KM) plotter website (http://kmplot.com) for TNBC (A, B), luminal B (C, D), HER2 (E, F), and luminal A (G, H). Luminal B, HER2, luminal A and Basal (for TNBC) breast cancer subgroups were obtained using the PAM50 classification setting. Median cutoff option was used. The obtained Hazard Ratio (HR) with 95% confidence interval and log-rank p-values are shown. ns (not significant), *p<0.05, ****p<0.0001.

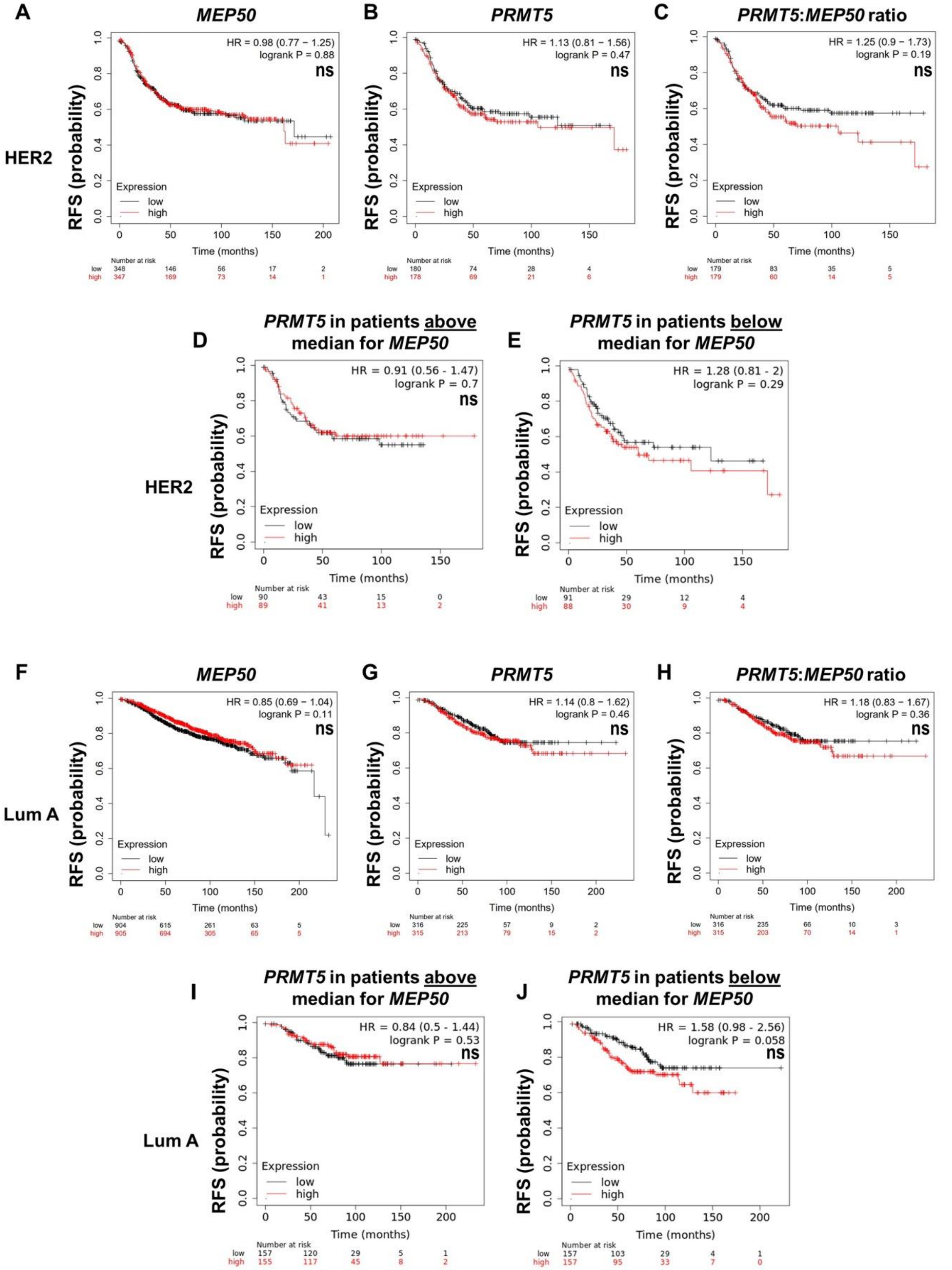

A
MEP50
HR = 0.98 (0.77 − 1.25)
logrank P = 0.88
ns
B
PRMT5
HR = 1.13 (0.81 − 1.56)
logrank P = 0.47
ns
C
PRMT5:MEP50 ratio
HR = 1.25 (0.9 − 1.73)
logrank P = 0.19
ns
HER2
RFS (probability)
Expression
low
high
Time (months)
Number at risk
low 348 146 56 17 2
high 347 169 73 14 1
low 180 74 28 4
high 178 69 21 6
low 179 83 35 5
high 179 60 14 5
D
PRMT5 in patients above median for MEP50
HR = 0.91 (0.56 - 1.47)
logrank P = 0.7
ns
E
PRMT5 in patients below median for MEP50
HR = 1.28 (0.81 - 2)
logrank P = 0.29
HER2
low 90 43 15 0
high 89 41 13 2
low 91 29 12 4
high 88 30 9 4
F
MEP50
HR = 0.85 (0.69 − 1.04)
logrank P = 0.11
ns
G
PRMT5
HR = 1.14 (0.8 − 1.62)
logrank P = 0.46
ns
H
PRMT5:MEP50 ratio
HR = 1.18 (0.83 − 1.67)
logrank P = 0.36
ns
Lum A
low 904 615 261 63 5
high 905 694 305 65 5
low 316 225 57 9 2
high 315 213 79 15 2
low 316 235 66 10 3
high 315 203 70 14 1
I
PRMT5 in patients above median for MEP50
HR = 0.84 (0.5 - 1.44)
logrank P = 0.53
ns
J
PRMT5 in patients below median for MEP50
HR = 1.58 (0.98 - 2.56)
logrank P = 0.058
ns
Lum A
low 157 120 29 5 1
high 155 117 45 8 2
low 157 103 29 4 1
high 157 95 33 7 0

Figure S3

Supplemental Figure S3. *MEP50* mRNA expression levels are not associated with RFS in HER2 and luminal A breast cancers. **A-C, F-G.** RFS based on *MEP50* mRNA, *PRMT5* mRNA expression or *PRMT5*:*MEP50* mRNA ratio were obtained from the Kaplan-Meier (KM) plotter website (http://kmplot.com) for HER2 (A, B, C) and luminal A (Lum A; F,G,H). **D,E,I,J.** RFS based on *PRMT5* mRNA expression within patients having either high (above median, left panel) or low (below median, right panel) *MEP50* mRNA expression (median cutoff). Data were obtained from the Kaplan-Meier (KM) plotter website (http://kmplot.com) for HER2 (D, E) and luminal A (Lum A; I, J). Of note, more patients were retrieved with MEP50 probe set compared to the PRMT5 probe set (see *Materials and Methods* section). The obtained Hazard Ratio (HR) with 95% confidence interval and log-rank p-values are shown. ns (not significant).

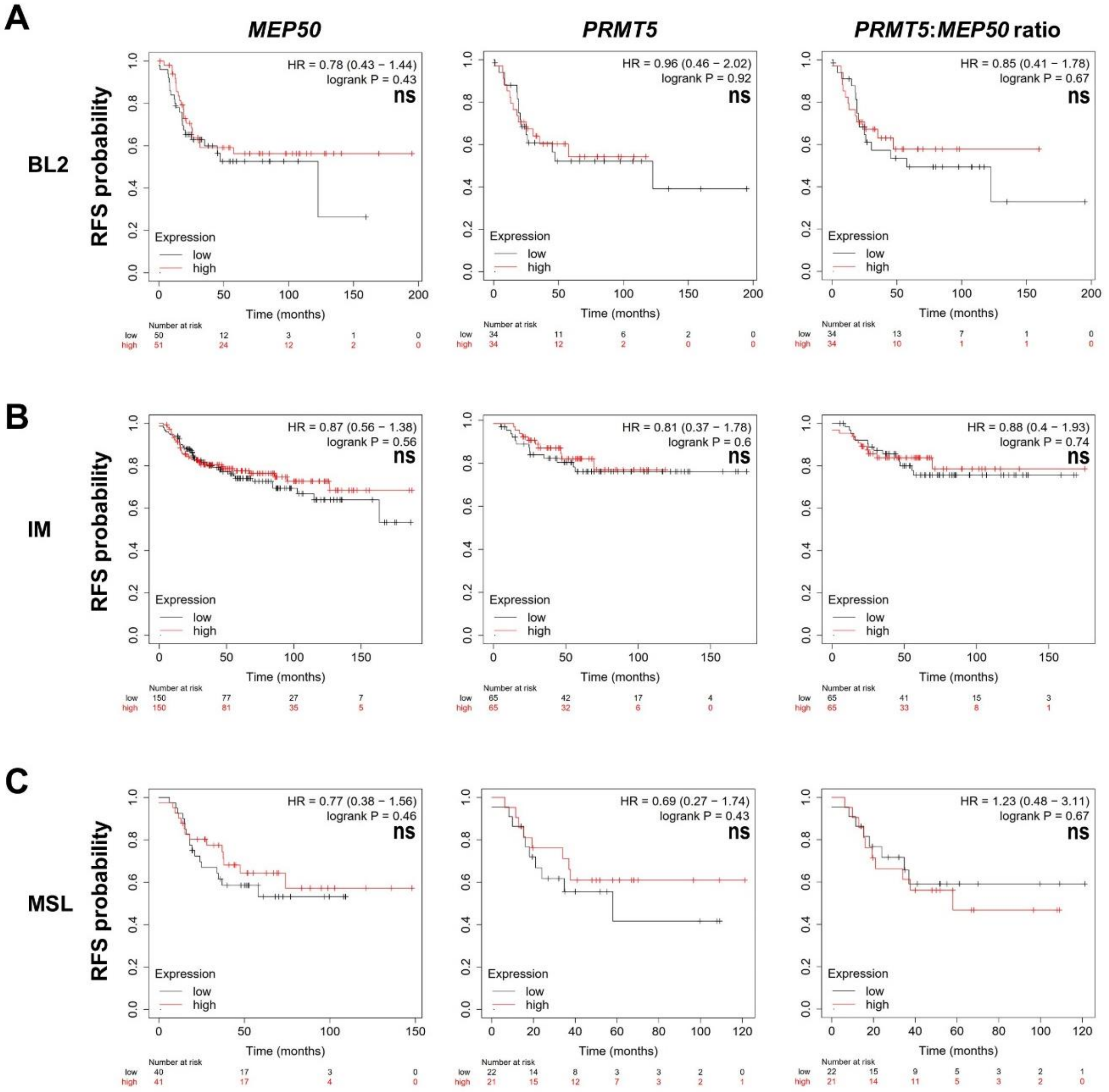

Supplemental Figure S4. *PRMT5*:*MEP50* mRNA ratio is not associated with RFS in BL2, IM and MSL TNBC subtypes. **A, B, C.** RFS based on *MEP50* or *PRMT5* mRNA expression or *PRMT5*:*MEP50* mRNA ratio were obtained from the Kaplan-Meier (KM) plotter website (http://kmplot.com) for basal-like 2 (BL2; A), immunomodulatory (IM; B) and mesenchymal stem-like (MSL; C) TNBC subtypes. Of note, more patients were retrieved with MEP50 probe set compared to the PRMT5 probe set (see *Materials and Methods* section). The obtained Hazard Ratio (HR) with 95% confidence interval and log-rank p-values are shown. ns (not significant).

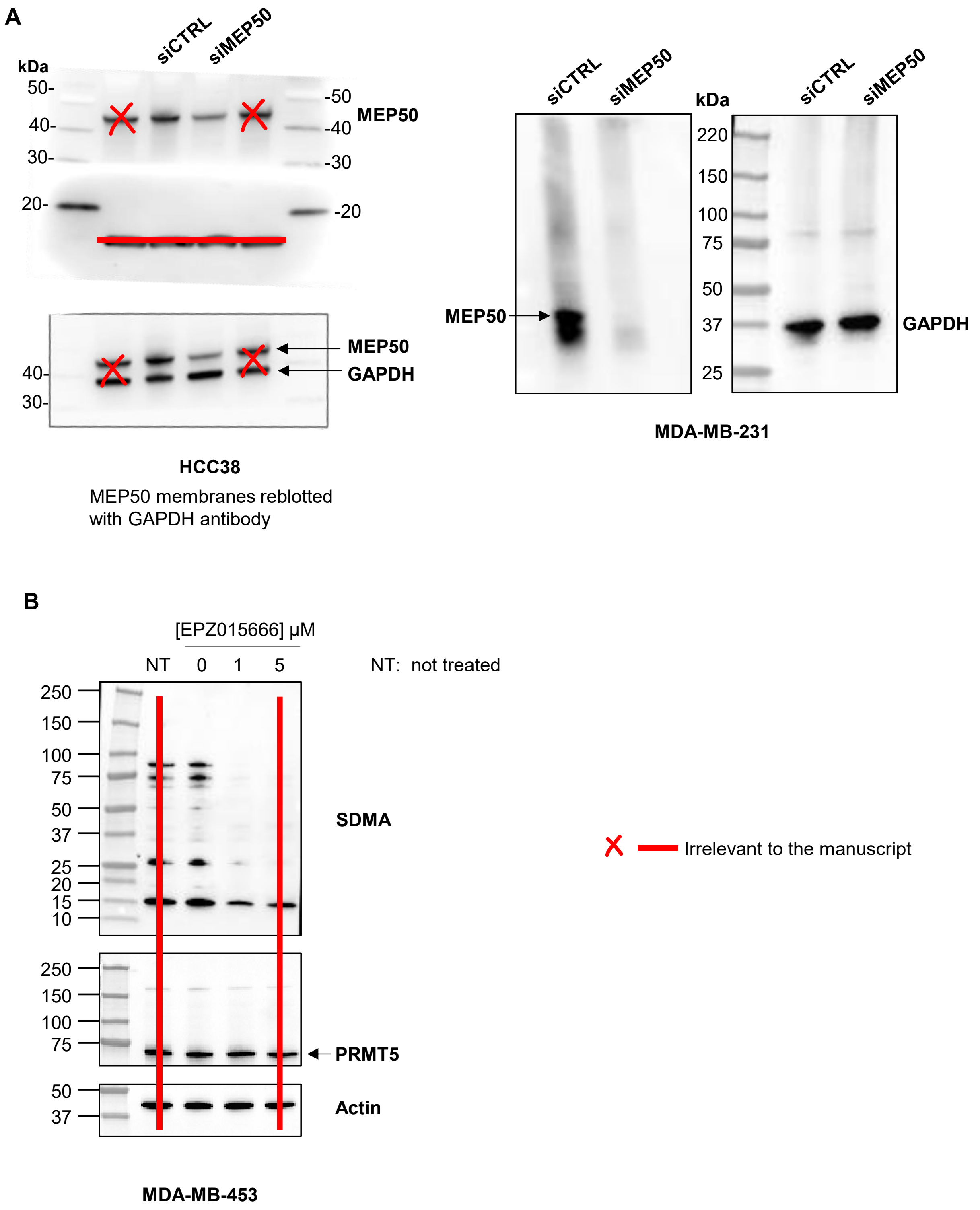

Supplemental Figure S5. Uncropped membranes of western blots corresponding to Figure 4 (A) and Figure 7A (B). Red crosses and dashes are irrelevant to the manuscript.

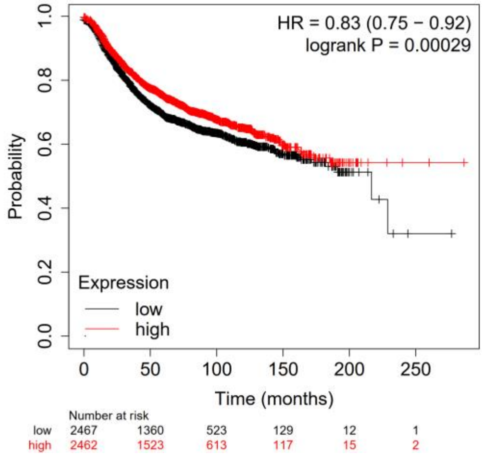

Supplemental Figure S6. High *MEP50* mRNA expression is associated with a better prognosis in all breast cancers. Recurrence-free survival (RFS) based on *MEP50* mRNA expression was obtained from the Kaplan-Meier (KM) plotter website (http://kmplot.com) for breast cancers, including all subgroups of breast cancer. The obtained Hazard Ratio (HR) with 95% confidence interval and log-rank p-values are shown.
